# Repurposing therapeutics for COVID-19: Rapid prediction of commercially available drugs through machine learning and docking

**DOI:** 10.1371/journal.pone.0241543

**Published:** 2020-11-12

**Authors:** Sovesh Mohapatra, Prathul Nath, Manisha Chatterjee, Neeladrisingha Das, Deepjyoti Kalita, Partha Roy, Soumitra Satapathi

**Affiliations:** 1 Department of Physics, Indian Institute of Technology, Roorkee, Haridwar, Uttarakhand, India; 2 Department of Pharmacology, Subharti Medical College, Meerut, Uttar Pradesh, India; 3 Department of Biotechnology, Indian Institute of Technology Roorkee, Roorkee, Uttarakhand, India; 4 Department of Microbiology, All India Institute of Medical Science, Rishikesh, Uttarakhand, India; 5 Centre of Nanotechnology, Indian Institute of Technology, Roorkee, Haridwar, Uttarakhand, India; Biotechnology HPC Software Applications Institute (BHSAI), UNITED STATES

## Abstract

**Background:**

The outbreak of the novel coronavirus disease COVID-19, caused by the SARS-CoV-2 virus has spread rapidly around the globe during the past 3 months. As the virus infected cases and mortality rate of this disease is increasing exponentially, scientists and researchers all over the world are relentlessly working to understand this new virus along with possible treatment regimens by discovering active therapeutic agents and vaccines. So, there is an urgent requirement of new and effective medications that can treat the disease caused by SARS-CoV-2.

**Methods and findings:**

We perform the study of drugs that are already available in the market and being used for other diseases to accelerate clinical recovery, in other words repurposing of existing drugs. The vast complexity in drug design and protocols regarding clinical trials often prohibit developing various new drug combinations for this epidemic disease in a limited time. Recently, remarkable improvements in computational power coupled with advancements in Machine Learning (ML) technology have been utilized to revolutionize the drug development process. Consequently, a detailed study using ML for the repurposing of therapeutic agents is urgently required. Here, we report the ML model based on the Naive Bayes algorithm, which has an accuracy of around 73% to predict the drugs that could be used for the treatment of COVID-19. Our study predicts around ten FDA approved commercial drugs that can be used for repurposing. Among all, we found that 3 of the drugs fulfils the criterions well among which the antiretroviral drug Amprenavir (DrugBank ID–DB00701) would probably be the most effective drug based on the selected criterions.

**Conclusions:**

Our study can help clinical scientists in being more selective in identifying and testing the therapeutic agents for COVID-19 treatment. The ML based approach for drug discovery as reported here can be a futuristic smart drug designing strategy for community applications.

## Introduction

The recent outbreak of novel Coronavirus disease (COVID-19) is now considered to be a pandemic threat to the global population [1–3]. Coronaviruses belong to a family of viruses mainly found in animals but with the recent outbreak, they have transmitted to humans. The new Coronavirus, 2019-nCoV is termed as severe acute respiratory syndrome-related Coronavirus SARS-CoV-2 [4–9] which has now affected more than 200 countries with over 25,326,924 cases confirmed and 8,49,060 deaths reported all over the world [as on 30^th^ August 2020]. This could potentially bring major challenges to global healthcare and disastrous effect on the global economy if the virus is not contained within a few months [10]. The common symptoms include cough, fever, shortness of breath, fatigue etc which makes it confusing for the patients to differentiate the symptoms with that of the typical cold and flu [10–13]. Reports suggest that the virus is transmitted through body fluids of the infected patients, especially when in contact and while sneezing even though exact reasons are not known. Unfortunately, no drugs have been approved by regulatory agencies to treat SARS-CoV-2 infection until now. Efforts are ongoing on war footing to find the effective drug and vaccine to treat this pandemic.

Coronaviruses are classified into four classes designated as alpha, beta, gamma, and delta [14]. The beta Coronavirus class includes severe acute respiratory syndrome virus (SARS-CoV), Middle East respiratory syndrome virus (MERS-CoV), and the COVID-19 virus (SARS-CoV-2). Coronaviruses are found to be considerably large viruses with a single-stranded positive-sense RNA genome encapsulated inside a membrane envelope having proteins appearing like spikes protruding from their surface. These spikes adheres onto human cells, through certain receptors on target cells, after which undergoes a structural change that lets the viral membrane fuse with the cell membrane. The viral genes then enter the host cell, producing more viruses. Recent studies show that, like the virus responsible for 2002 SARS outbreak, SARS-CoV-2 spikes also bind to receptors on the human cell surface called angiotensin-converting enzyme 2 (ACE2) [15]. Like SARS-CoV and MERS-CoV, SARS-CoV-2 also attacks the lower respiratory system causing viral pneumonia. However, there are also reports that it could affect the gastrointestinal system, heart, kidney, liver, and central nervous system resulting in multiple organ failure [16]. Compiling the medical reports and data available from the patients, SARSCoV-2 is found to be more transmissible/contagious than SARS-CoV [17].

Rapid development of computer aided technology like ML based on Artificial Intelligence (AI) can help accelerate the drug development process for different diseases [18–20]. The advantage of AI approaches like ML is that they can be applied to learn from examples and build predictive models even when our understanding of the underlying biological processes is limited, or when computational simulations based on fundamental physical models are too expensive to be carried away. Another advantage of ML is to automatically learn to identify complex patterns that categorize sets from input data and thereby make intelligent decisions based on independent datasets [21]. ML can accurately predict drug-target interactions as an enormous amount of complex information by studying hydrophobic interactions, ionic interactions, hydrogen bonding, van der Waals forces, etc. between molecules. Bioactivity datasets which are available from the numerous high throughput screens deliver useful means for machine learning classifiers as they contain binary information (active/inactive) as well as numerical values to classify different compounds under consideration [22, 23]. Such a huge number of datasets available on biological activities of molecules, derived from high throughput screens now allows to create predictive computational models.

In this study, we have applied a machine learning approach to predict several new potential drugs for the treatment of SARS-CoV-2 and validated the predicted drugs. Initially, we have trained our model with the inhibitors of the *SARS Coronavirus 3C-like Protease*. The FDA approved drugs are only taken from the Drug bank as a test model to predict the new drugs. These new drugs are again validated using a docking method to ensure that the drugs match with the same active site on the protein. A ranked list of drugs based on energy value is given that can be tested experimentally. Our study hypothesizes that the commercial FDA approved antiretroviral drug Amprenavir may be a potential candidate requiring to limit viral recognition of host cells or disrupt host-virus interactions thus requiring further clinical trial. Recently, several computational studies have reported the potential of FDA approved drugs for Covid-19. Rodriques et al reported a study on potential drugs for Covid-19, which showed that Atazanavir could dock in to the active site of SARS-CoV-2 Mpro with greater strength compared to Lopinavir which is also a HIV protease inhibitor [24]. Beck et al. published a study based on deep learning model predicted Atazanavir having high inhibitory potency against SARS-CoV-2 among other FDA approved drugs [25]. Ekin et al. also reported on repurposing of drugs for covid-19 based on polypharmacology using ‘molecular and biological signature’ which is guided by artificial intelligence reported several potential drugs including Atazanavir which can be repurposed for covid-19 [26]. They have compared several FDA approved drugs with matching scores which could further accelerate studies on SARS-CoV-2 drug discovery [26]. Another latest study published by Arshad et al. predicted some of the FDA approved drugs after evaluating existing in vitro anti-SARS-CoV-2 data, compiling all reports available [27]. In the present study, we have used a computationally less involved Naive Bayes algorithm to successfully train and predict some FDA approved drugs. We found 10 such drugs and Amprenavir has lowest global energy value which can a potential candidate for further clinical study.

## Methods

### Preparation of dataset

In the present study, the compounds of the dataset are tested in the cell based system using plate readers and then their results are stored as Bioassay Dataset in the Pub chem. This dataset of PubChem Bioassay assigned AID 1706 contains around 290893 compounds as one activity set and they are the inhibitors of *SARS Coronavirus 3C-like Protease* in the cells. This dataset is stored in the section Bioassay of PubChem database of National Centre for Biotechnology Information (NCBI), and they have the identification AID number as AID 1706 [23]. This corresponding bioassay belongs to the Scripps Research Institute Molecular Screening Center of replication in *SARS Coronavirus 3C-like Protease* in the cells. The compounds are classified under three distinct categories as actives, inactives and inconclusive. Compounds that inhibit luminescence activity may kill *SARS Coronavirus*, inhibit *SARS Coronavirus* invasion or inhibit development of the parasite within the host cell and hence these are classified under the active section and the compounds which do not show effectiveness are classified under inactive section. These complete datasets were downloaded in the form of SDF (Structure Data File) from the PubChem Database.

**Fig 1:**
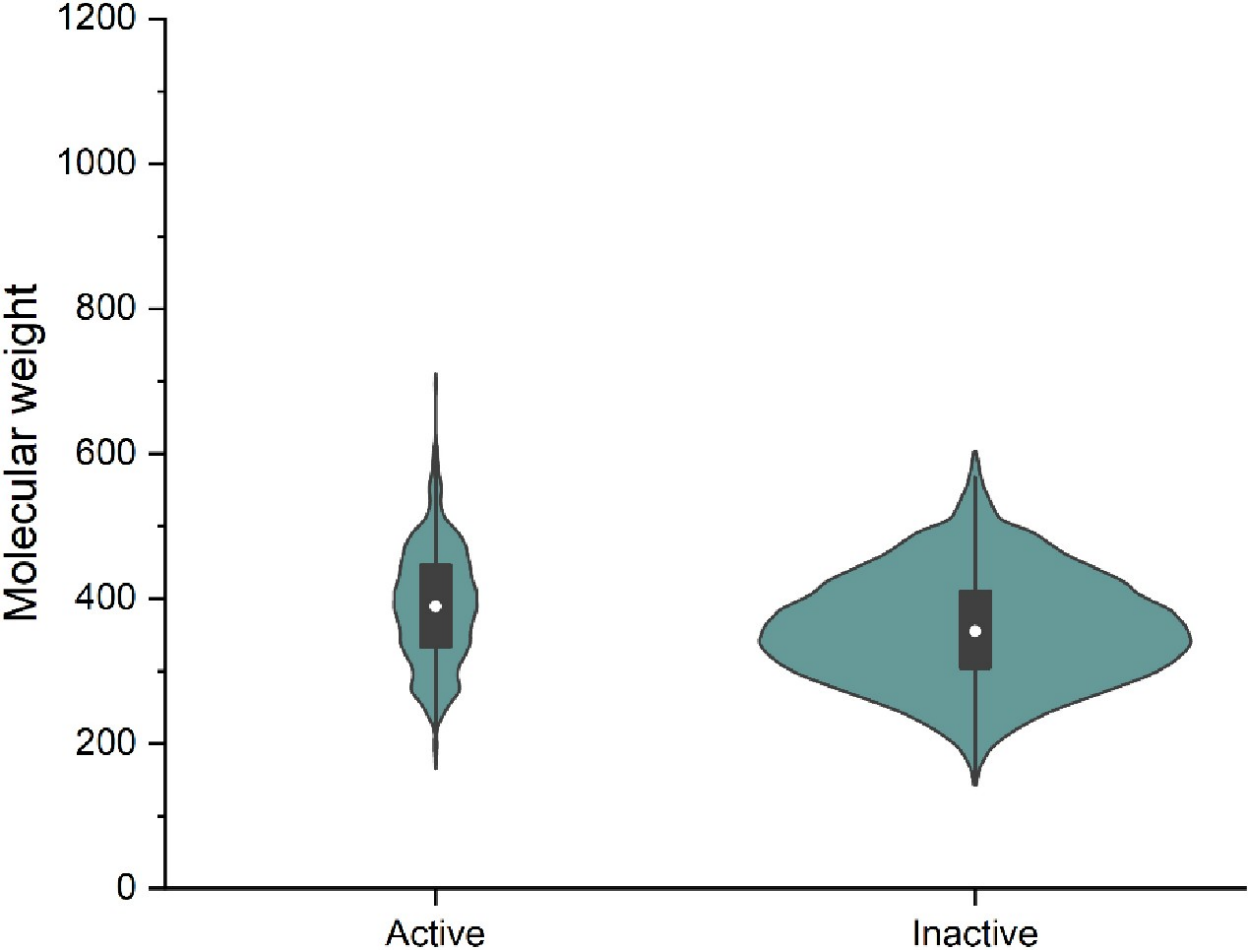
Representation of the variation in the molecular weights of the considered active and inactive pharmacophore fingerprints.

The Drug Bank is an online database which contains detailed data about various medications [24]. Today, it is being widely used to facilitate in silico drug target discovery, drug design, drug docking or screening, drug metabolism prediction, drug interaction prediction and for general pharmaceutical education. This database of more than 4900 Drugs is categorized into many different types as Trial stages Drugs, Approved Drugs and Withdrawn Drugs. In this database, more than 45% of drugs are approved for various medication purposes [28]. In this research, we have focused only on the FDA approved drugs for repurposing purpose which are around 2388 with the intention that it will minimize clinical trial in the present situation. These drugs were downloaded in the form of SDFs and after processing, the descriptions generated were taken as the test model for developing the train model which was made on the basis of a database containing the inhibitors of the *SARS Coronavirus*. The developed model has predicted few of the potential drugs. Fig 1 represents the variation in the molecular weights of the considered active and inactive pharmacophore fingerprints.

### Processing dataset

Since the datasets are present in the form of SDFs, we have generated the attributes present in the SDFs. First, the information present in the SDFs are generated as CSV files which are used as the training dataset and test dataset for preparing the ML models. These CSV files containing both the actives and inactive points are split into 80% as training dataset and 20% as test dataset. This entire splitting process was random. This process is done by self-written python code to split as per the conditions.

### Classification algorithm

We have used Machine learning (ML) model to the selected dataset from the PubChem which was considered as inhibitors and tested against the drugs from the Drug Bank to find more suitable drugs for the Covid-19 [29]. Using ML, we have implemented the classification algorithms as described below.

The classification is a type of supervised learning in which the computer system can learn from the dataset which contains the detail and practical results. The algorithmic procedure of the classification is to assign an input value according to the description in the datasets [30]. So, for this, it requires a mathematical classifier that can assign specific class (active and inactive) labels to instances defined by the attributes. In this process, the training model is made to learn using dataset where the classification is already assigned and on the basis of which it is able to run on different datasets to classify them according to the present instances. In this study, we have compared the results from the classifier that is Naive Bayes classification algorithm.

Naive Bayesian classification algorithm is a simple and elegant approach by assuming that its classification attributes are independent and they don't have any correlation with each other [31]. It is a type of classifier that depends on Bayes' hypothesis. Naive Bayes does work best in two cases: complete independent feature (as expected) and functionally dependent features (as expected) and is a widely tested method for probabilistic induction.

This algorithm is more useful than any other induction algorithms because of its computation speed and reliability [29]. Also, when the analysis of several other algorithms were done, it was found that the classification algorithms like Randome Forest, SMO, etc were getting overfit on the provided dataset. It is based on the analysis of several other algorithms which when used, showed that they overfit on the dataset. The reason of overfitting can be various but the most significant one is related to the dataset which contains binary codes and few other required information only. Hence, it can be useful for both the binary classification as well as multi-classification.

### Training model

The training model is based on the collected pharmacophore fingerprints that the dataset contains and to divide it into the testing and training model, we have used 80% of the entire dataset as the training model whereas the rest 20% is taken as the test model or set. The dataset is completely classified from where the computer learns and finds the relations among various attributes. The cross-validation is used along with the algorithm to train the model. In this case, we have used 10-fold and it is chosen as per the size of the dataset [32, 33].

Generally, the datasets containing binary classification based on several attributes are imbalanced. We observe the similar trend here. These imbalanced datasets are not possible to be handled by the normal classifiers since they give importance to each of the attributes equally which could lead to misclassification errors. This can decrease the accuracy of the dataset for the trained model [34, 35]. Therefore, we have used the misclassification cost where the trained model becomes cost sensitive and able to find the lowest expected cost. This approach is actually much randomized because it neither depends upon the number of attributes nor on the minority class ration; rather it depends on the base classifier [36, 37].

Here, we had two methods to introduce the misclassification cost with the imbalance dataset. The first method is to classify the algorithm into the cost-sensitive one and proceed with the rest settings [38]. The other is the use of a wrapper, which helps in the base classifiers into cost sensitive ones.

We have used Naive Bayes classifier which uses the cost insensitive algorithm to predict the probability estimations of the test instances and then using this it predicts class labels for the examples of the test dataset. In our report, we have classified our datasets into two classes i.e. active and inactive. So, we used the 2X2 matrix which is generally used for the binary classification. In the matrix sections, we can find True Positives (active classified as active), False Positives (Inactive classified as active), False Negatives (active classified as inactive) and True Negatives (inactive classified as inactive). In this case, the percent of False Negatives are more important than the percent of False Positives and the upper limit for False Positives were set to 20% [34, 38]. In this process, we increase the misclassification up to the set percent which also helps in the increasing of the True Positives.

Since, the actives are very less in number, we have replicated them to around 100–110 times to match it with the inactives and make the model less biased.

### Independent validation

There are various methods for the validation of the binary classifiers. The True Positive Rate is the ratio of the actual actives to the predicted positives and this can be obtained as (TP/TP+FN). The False Positive Rate is the ratio of the predicted false actives to actual inactives and this can be obtained as (FP/TN+FP). Accuracy shows the model’s performance relative to the real values and this can be calculated as (TN+TP/TN+TP+FP+FN). The Sensitivity shows the model’s ability to identify the positive results and this is calculated as (TP/FN+TP) and the Specificity shows the model’s ability to identify the negative results and this is calculated as (TN/TN+FP). A model with high specificity and sensitivity has a low error rate. The Balanced Classification Rate (BCR) is the mean of the sensitivity and specificity, which provides the accuracy of the model applied on the imbalanced dataset. This BCR can be calculated as 0.5*(specificity+sensitivity).

Apart from the BCR, the Mathews Correlation Coefficient (MCC) is also used whose range varies from -1 to 1. We have also found the F Score which gives a better idea about the model. The Receiver Operating Characteristic (ROC) curve is the visualization of the ratio of FPR to TPR. In this case, the FPR and TPR are placed on the x- and y-axis respectively. The Area under curve shows the probability prediction of the classifier and its ability to classify the randomly chosen instance into the correct class.

After the preparation of the training model, then the rest 20% of the dataset that was kept from the original dataset was run against the training model. Finally, this gave the test performance with an accuracy of around 72.999% or 73%.

### Docking of the predicted drugs

Around 178 drugs were predicted by our ML model which can be effective for the treatment of diseases caused by *SARS-Cov-2*. The docking of the drugs was done with SARS-Cov 3C-like protease. The PDB structure of SARS-CoV 3C-like protease was retrieved from Protein data bank (PDB ID: 3VB7). For preparation of the optimized protein structure for docking, all the water bodies as well as previously attached ligands were removed. However, the drugs were not being prepared; instead, we used the already available drugs from the DrugBank. All the docking experiments were carried out using the patchdock server (https://bioinfo3d.cs.tau.ac.il/PatchDock/). While providing the input parameters in the patchdock server, the active sites were provided as inputs for targeted docking. Information on the active sites were collected from Chuck et. al., 2013 [39]. There are no available drugs as of now, since the epidemic has just recently accelerated to over 25,326,924 cases [As of 30^th^ August, 2020].

The predicted compounds with above 95% of confidence were docked using patchdock web server.

## Results

**Fig 2:**
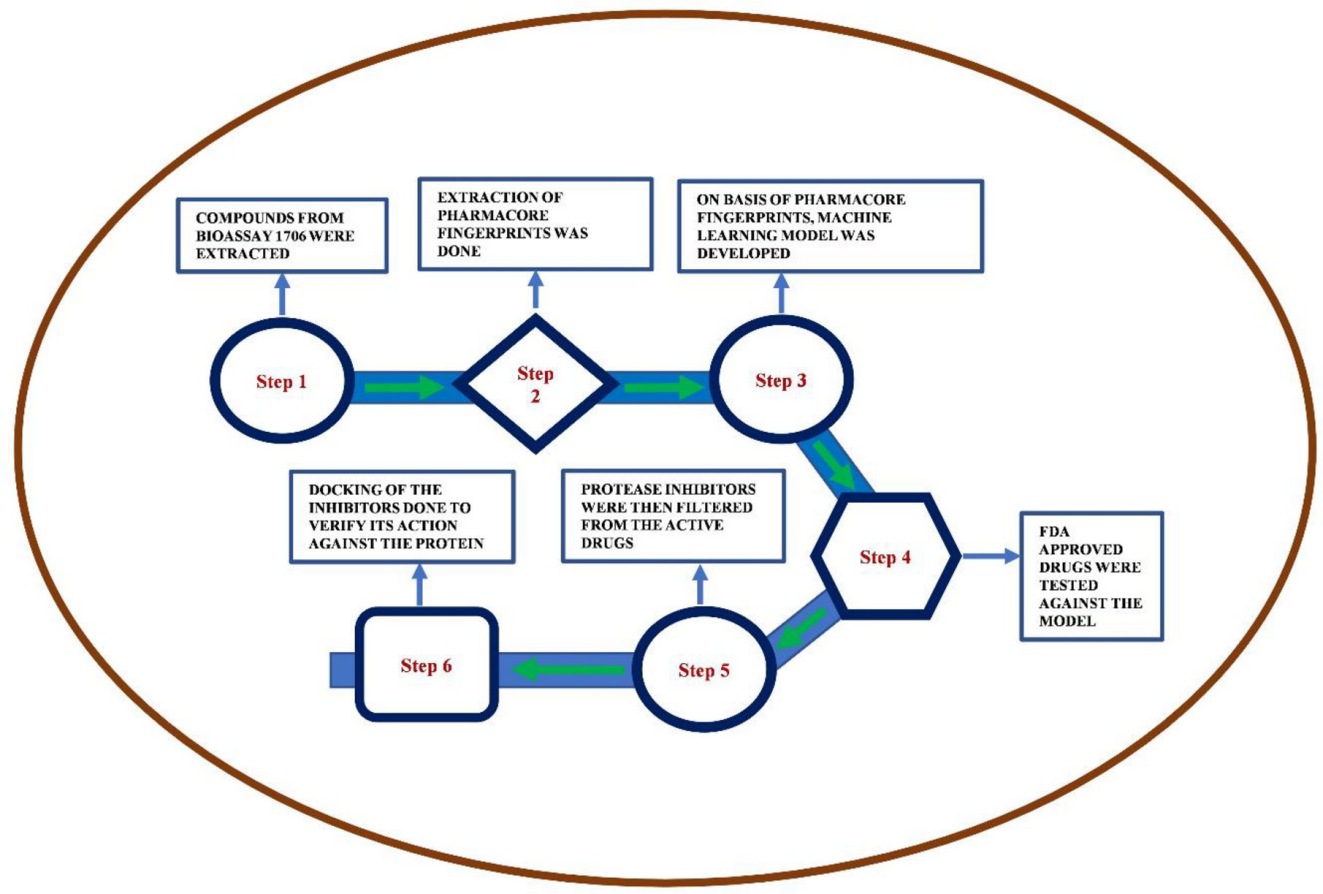
Flowchart of the machine learning based approach to predict FDA approved drugs for COVID-19.

Here, we have at first taken the inhibitors of SARS-CoV-2, which doesn’t allow them to replicate in the host. These are screened and collected in the bioassay AID 1706 which were used as the main component for the modeling of the training model using ML. The 914 attributes were taken under consideration for more than 200,000 compounds. We have not used unsupervised learning to filter out the dataset, because it would have made the dataset much weaker. As mentioned in the method section, we have used a classifying algorithm to train the model and the best among them was further used for the testing and predicting the drugs from the Drug Bank. The schematic of the process is shown in Fig 2.

We have used the Naive Bayes against the dataset. The test performance has shown accuracy of approximately 72.999% whereas the training model had an accuracy of 72.67% Along with that, the model has a True Positive accuracy rate of 73% and False Positive Accuracy of 50.7%. With this model, we have used the drugs from the Drug Bank to get predicted for the identification of the potential drugs which can be used for the treatment of disease caused by *SARS Coronavirus*. Along with that the model has predicted 34754 True Positives and 3904 True Negatives.

**Fig 3:**
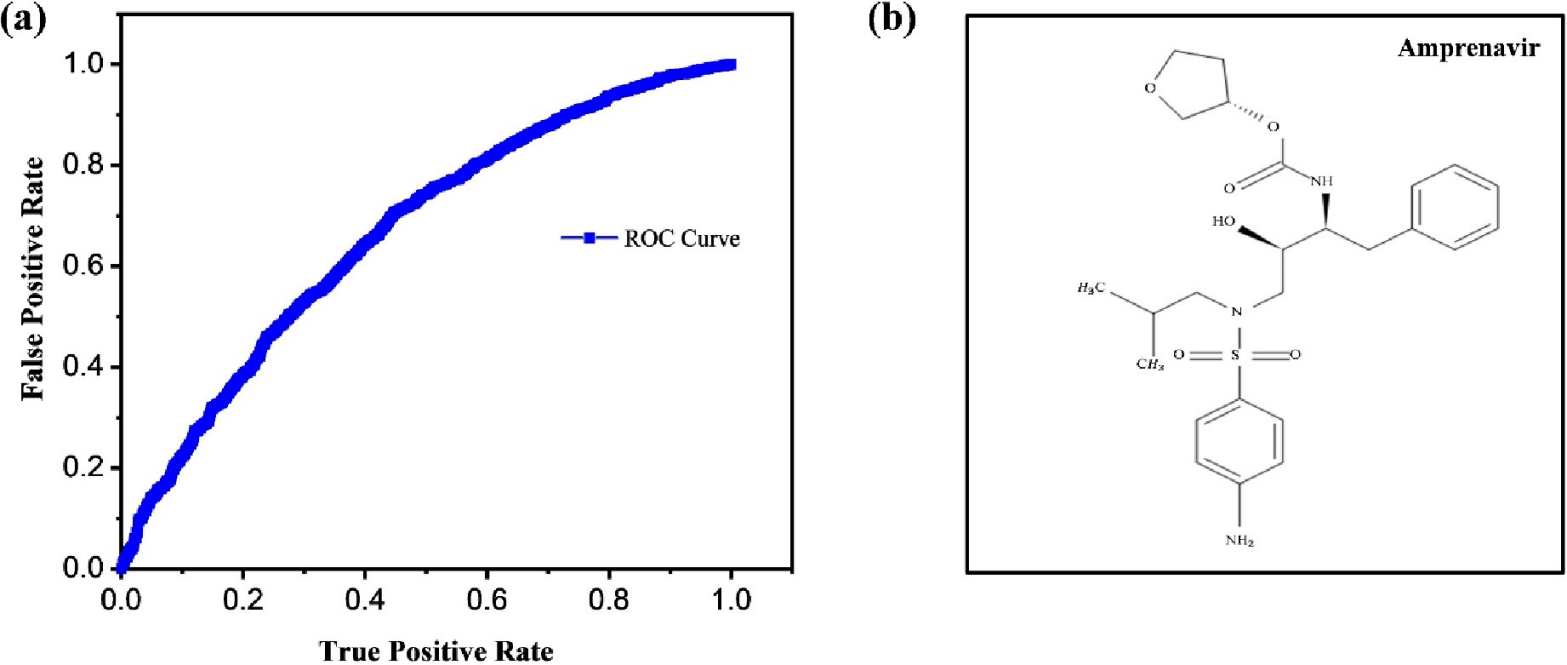
(a) The comparison of the false positive rate and true positive rate for all algorithms used in case of preparation of machine learning models (b) Chemical structure of the Atazanavir.

The Naive Bayes has 0.194 MCC and BCR i.e., 76.69%. The AUC-ROC value of the ML model is 0.666 or 0.67 Along with the other results, the F Score is 0.768, which presents that the model is fairly good to predict the potential drugs for COVID-19. As per the independent validation, the algorithm with the lowest possible False Positives and highest possible True Positives can be considered to be the most effective model for the prediction of the drugs from the Drug Bank. In all the cases, the compounds for the False Positives were set to 20%. The comparison of the False Positive Rate and True Positive Rate for all algorithms used in the case of preparation of ML models are shown in Fig 3A. When these results are compared with the rest of the dataset, it is found to be way better because it has satisfied both criteria and the rest of the algorithms has not reached the mark that has been achieved by Naive Bayes.

The model created with Naive Bayes algorithm predicted around 471 drugs out of all the 2388 approved drugs. These 471 drugs contain all the drugs which may be effective for the treatment of disease caused by SARS-Cov-2. The selection of these drugs is based on the confidence level of the model. The model has predicted the 471 drugs over a range of confidence levels starting from 51% - 98%. However, we have used the drugs for the processing to the docking stages which had a confidence level of more than 90%. After this step, we were left with around 28 drugs which were again docked with the potential protein molecule. Hence, we have suggested the top 10 of them considering both the ML accuracy and the docking result. The docking results have been used for binding energy prediction and its effectiveness to bind with the compound. The results are shown in Table 1.

**Table 1:** Global energy value for predicted drugs.

Drug Bank ID	Name of Drugs	Global Energy	Attractive VdW	Repulsive VdW	ACE
DB00701	Amprenavir	-58.20	-23.71	10.20	-19.77
DB01319	Fosamprenavir	-56.52	-24.16	9.99	-18.25
DB00224	Indinavir	-54.80	-23.28	3.63	-16.7
DB01232	Saquinavir	-54.49	-25.41	11.86	-18.20
DB01264	Darunavir	-54.31	-24.72	5.92	-15.99
DB00503	Ritonavir	-52.39	-22.04	11.48	-20.31
DB09297	Paritaprevir	-48.47	-22.87	7.59	-14.76
DB01601	Lopinavir	-46.04	-21.26	3.58	-13.13
DB01072	Atazanavir	-45.19	-22.02	15.61	-17.63
DB00932	Tipranavir	-36.68	-16.99	7.46	-12.64

Based on the docking study, we identified 10 possible drugs namely Amprenavir (DrugBank ID–DB00701), Fosamprenavir (Drug Bank ID: DB01319), Indinavir (Drug Bank ID: DB00224), Saquinavir (Drug Bank ID: DB01232), Darunavir (Drug Bank ID: DB01264), Ritonavir (Drug Bank ID: DB00503), Paritaprevir (Drug Bank ID: DB09297), Lopinavir (Drug Bank ID: DB01601), Atazanavir (Drug Bank ID: DB01072) and Tipranavir (Drug Bank ID: DB00932) for the treatment of novel SARS Coronavirus. The chemical structure of Amprenavir is shown in Fig 3B. Chemical structures of remaining 9 drugs are provided (S4–S12 Figs in S1 File).

**Fig 4:**
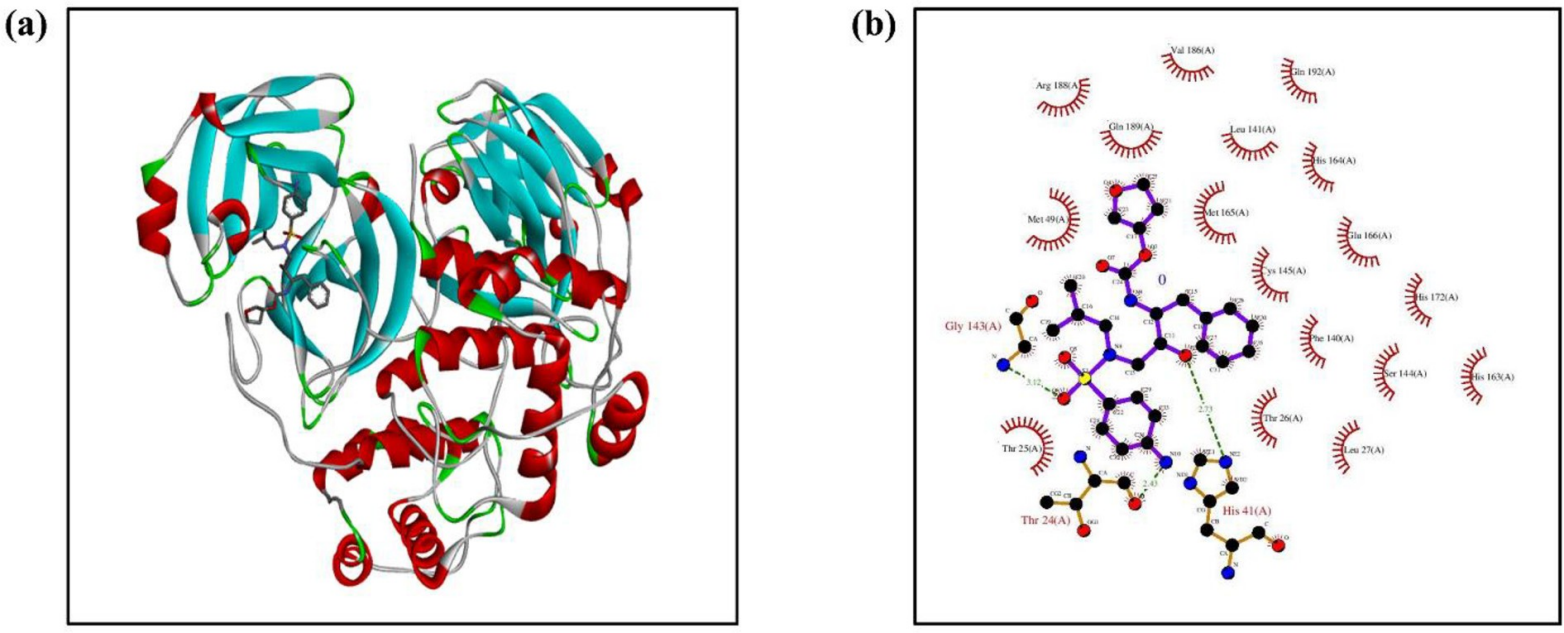
(a) Docking pose of SARS-CoV 3C- like Protease complexed with Amprenavir, (b) ligplot analysis showing possible bonds between protein and ligand.

In order to evaluate the similarity between the protein selected i.e. SARS COV 3C like protease and SARS COV2 protease, we evaluated a protein BLAST using NCBI’s server (https://blast.ncbi.nlm.nih.gov/Blast.cgi). The blast was done between FASTA sequence of PDB: 3VB7_B and PDB: 6M0K_A. It was observed that there was a 96% similarity in both the structures (S1 and S2 Figs in S1 File). Initially, the structure was optimized by removing water molecules and other ligands. Structure of all the ligands were retrieved from PubChem (https://pubchem.ncbi.nlm.nih.gov/). The docking experiment was done with the patchdock server (https://bioinfo3d.cs.tau.ac.il/PatchDock/). The results refinement and energy calculation was performed as per the algorithm used in the Firedock server. In server, the final ranking was performed to identify the near-native refined solution. The ranking was based on various binding energy functions that includes a variety of energy terms: van der Waals interactions, partial electrostatics, desolvation energy (atomic contact energy, ACE), hydrogen and disulfide bonds, p-stacking and aliphatic interactions and rotamer’s probabilities [40]. The result having minimum global energy was taken into consideration. Out of all 10 drugs predicted, Amprenavir (DrugBank ID–DB00701) (Fig 4A and 4B) has shown the minimum global energy. To access the ligplots and detailed protein interactions, the solution were further analysed with PDBsum (http://www.ebi.ac.uk/thornton-srv/databases/cgi-bin/pdbsum/GetPage.pl?pdbcode=index.html). The active sites of the selected protein was analysed from the source information i.e. from which the protein structure was experimentally obtained [39]. In the original study (where the authors obtained the enzyme-inhibitor complexes crystal), the binding pocket involved with the M4Z (ligand) are- Cys145 (covalent interaction); with His163, His164, Glu166, Thr190 and Phe140 via H-bond interactions; and with Gln189 and His41 via hydrophobic interactions. The same study also claims that the protein catalytic site (His41 and Cys145) and the protease inhibitors may probably bind with two of the any of these residues. In order to support this fact, we also predicted the binding pocket of the protein (PDB ID: 3VB7) by using CLASTp web server (http://sts.bioe.uic.edu/castp/index.html?3vb7). The web server follows the analysis of binding pockets and cavities based on the recent theorectical and algorithmic results of computational geometry [41]. The information on the binding pocket positions and amino acids sequence in the protein are provided in the S3a and S3b Fig in S1 File. In brief, as per the binding pocket prediction of the CLASTp, the important residues of the binding pocket are–A:41, A:145, A:189 and A:248. Fig 4B shows the ligplot analysis of our best result i.e interaction of SARS-CoV 3C- like Protease with Amprenavir. The result shows three conventional H-bonding between ligand and protein. One H-bond was observed between Thr (24) A of the protein & O 169 of the ligand. Similarly, the second one was between His (41) A & NE2 312. The third H- bond was observed betwwen Gly (143) A and N 1105. These H-bonds favoured a strong bonding between the two moieties. The bond lengths of the above three H-bonds are 2.43Å, 2.73Å and 3.12Å respectively. However, the binding pocket in this case involved- His (41) A, Thr (24) A, Gly (143) A via H-bonding and Thr (25) A, Thr (26) A, Cys (145) A, Met (165) A, Gln (189) A via non-bonded contacts. The protein structure (PDB ID 3VB7), has two catalytic sites and His (41) A is one among them. Interestingly, the binding of the drug Amprenavir to the the protein at His (41) A proves the capability of the drug to inhibit the protease and can be declared as a potent inhibitor.

## Discussion

In our study, we have taken only those drugs for docking purpose which showed protease inhibition activity. The docking of SARS protein with the approved drug Amprenavir (DrugBank ID–DB00701), has a minimum global energy of around -59.90 Kcal/mol among all the compounds. Inspite of the steric interactions at Ser121(B) and Pro122(B), the global energy of -59.90 Kcal/mol was quite favourable due to negative free energy and suitable bond lengths. These factors highlight its potential to inhibit novel SARS Coronavirus. Amprenavir is a small molecule antiretroviral drug (Fig 3B), usually sold under the brand names Invirase and Fortovase, used together with other medications to treat or prevent HIV/AIDS. It is an HIV protease inhibitor which acts as an analog of an HIV protease cleavage site. It is a highly specific inhibitor of HIV-1 and HIV-2 proteases. Fosamprenavir and Indinavir are also found to have quite favourable global energy and thereby they are also having potential to be effectively used to inhibit SARS coronavirus. Fosamprenavir is an antiretroviral drug commonly used for the treatment of HIV infection. It is quickly activated to amprenavir which is a potential HIV protease inhibitor. Ritonavir similarly used for the treatment of HIV infection as a combinational therapy and act as a pro-drug for HIV protease inhibitor. In this study, we highlight the best one out of the FDA approved drugs after the docking mechanism which is Amprenavir.

In addition to the docking of the above drug, we have also docked several other approved drugs available in the Drug Bank, which are predicted by our ML model with a confidence level of above 95% and also shows the activity of protease inhibition (S13–S17 Figs in S1 File). With reference to that, we have found that the other drugs predicted by the model with the inclusion of all the parameters taken under consideration can also be quite effective.

The rapid identification of active therapeutic agents against SARS-CoV-2 is a major challenge. Analyzing the available knowledge on their safety profiles, and in some cases, efficacy against other Coronaviruses and repurposing existing antiviral drugs is a potentially crucial short-term strategy to tackle COVID-19.

Under the current scenario, it takes more than 15 years to bring a drug from the investigational stages to market availability. It is because of the trial and error process or the so-called Edisonian Approach, where one keeps on analyzing several compounds to find the best possible one. These days, with the inclusion of digital medicine, this time span has been reduced to a great extent and studies are more approachable in a rational manner for the drug discovery process. Here, we have targeted the repurposed drugs towards the development of effective treatment of COVID-19 to speed up clinical trial. Recently, several work has been carried out to repurpose various FDA (Food and Drugs Administration) approved drugs against COVID-19. FDA already has approved various direct acting drugs against several other viruses. The structural similarilty of the SARS-CoV-2 with various other forms of viruses from the same family provided a hint for the further repurposing. In case of Hepatitis C Virus (HCV), drugs like Sofosfubir and Ribavirin are nucleotide derivatives and compete with physiological nucleotide for RdRp active site [42, 43] Additionally, as per the recent studies, if we compare the IC50 of Ribavirin in Dengue and Covid-19, the value is slightly higher for COVID-19 (109.5μM) than Dengue (8 μM) [44, 45]. Hence, the drugs can be repurposed but with certain higher concentrations. Since the pandemic grasped the whole wolrd, clinical trials of repurposing dirrent anti-HIV drugs and anti-malarial drugs were carried out. Interstingly, some of them have shown promising effect in the management of COVID-19 patients. Chloroquine has been used worldwide for more than 70 years, and is well kown for its anti-malarial activity. Recent evidences shows the apparent efficacy of the drugs in the management of COVID-19 patients [46, 47]. We have found that ML model created on the basis of the Naive Bayes algorithm is the most effective one with the accuracy of almost equal to 73%. The drugs predicted by this model is further verified by the docking process. We speculate that our predicted drugs show immense potential for treatment of the COVID-19.

Considering the ongoing efforts to prevent the spread of COVID-19 all over the world, we are optimistic that the outbreak may subside in a few months like SARS and MERS. However, the outbreak has stressed the urgent need for renewed efforts towards the development of broad-spectrum therapeutic agents to combat Coronaviruses which are repeatedly found to be a realistic threat of this century till now. Our this finding will provide a base for further enhanced drug discovery programs.

## Supporting information

S1 File (DOCX)
