# Supplementary material for: Repurposing therapeutics for COVID-19: Rapid prediction of commercially available drugs through machine learning and docking
Source: PLoS One. 2020 Nov 12;15(11):e0241543. doi: 10.1371/journal.pone.0241543 (PMC7660547; doi:10.1371/journal.pone.0241543)
Supplement: S1 File — (DOCX) [file pone.0241543.s001.docx]

Supplementary information


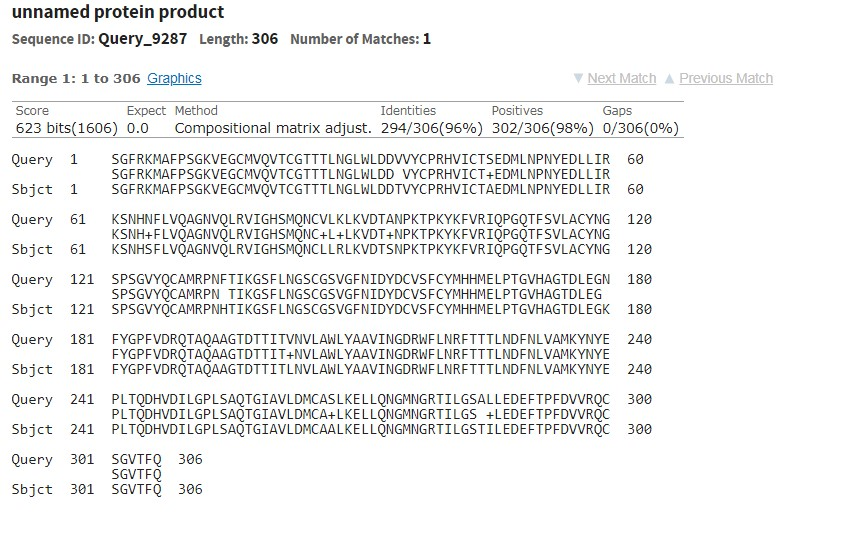


**Figure S1: Sequence alignment of both the proteins**

**
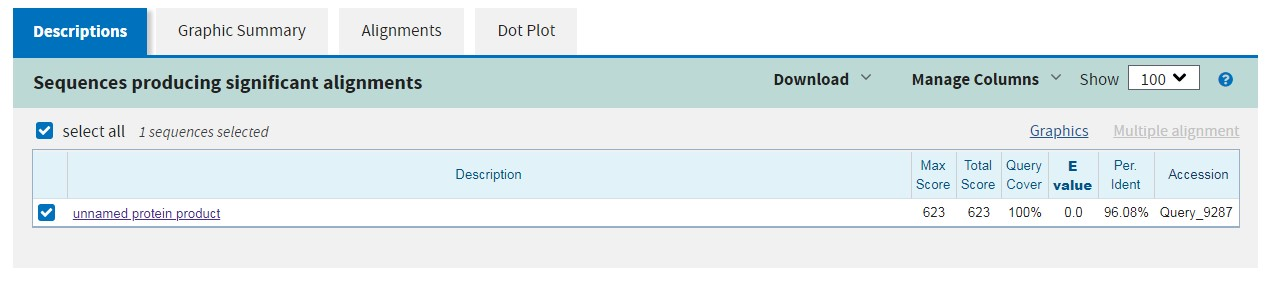
**

**Figure S2: Blast result of alignment between PDB: 3VB7_B and PDB: 6M0K_A**

**
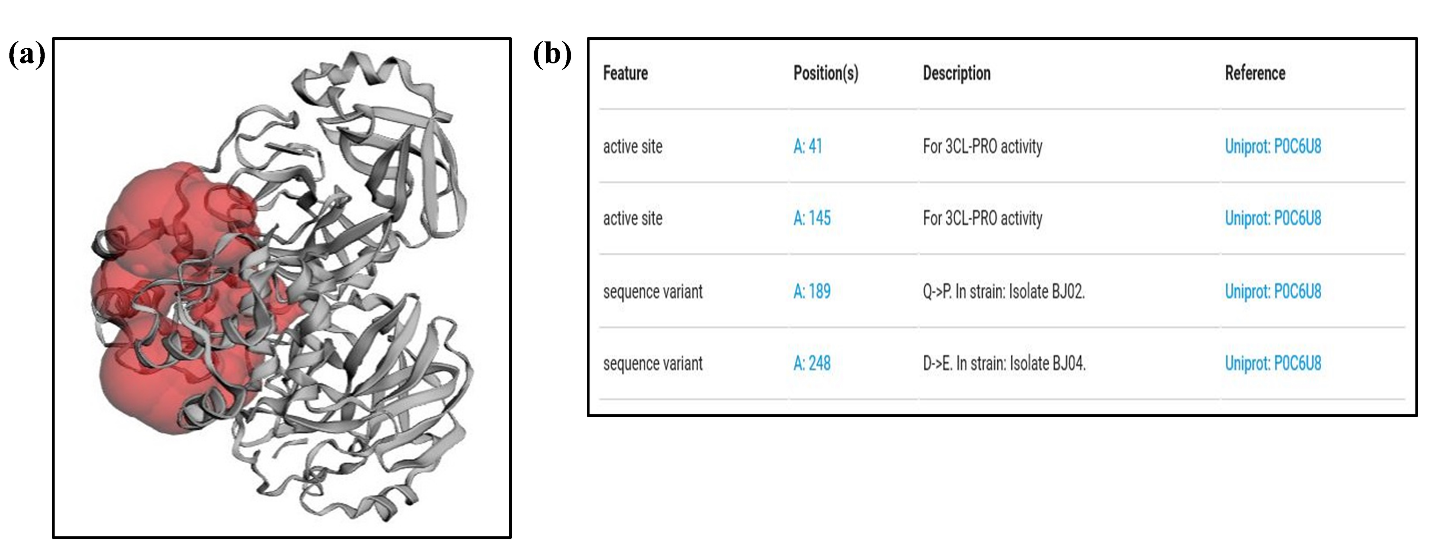
**

**Figure S3: Binding pocket estimation of SARS COV 3C protein by CLASTp**

**
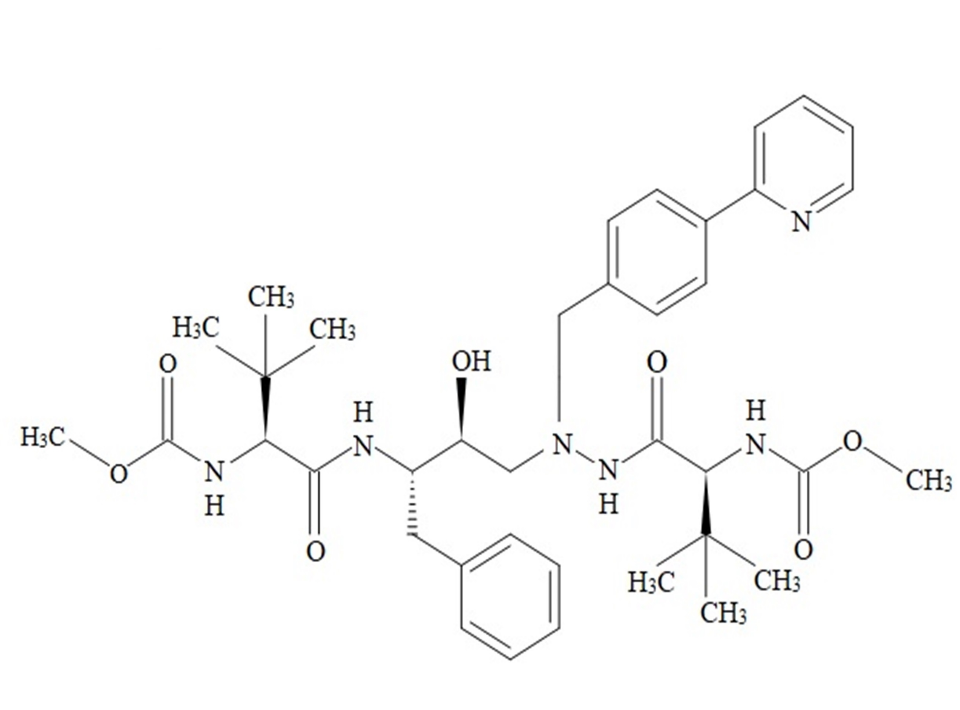
**

**Figure S4: Structure of Atazanavir**

**

**

**Figure S5: Structure of Darunavir**

**

**

**Figure S6: Structure of Fosamprenavir**

**
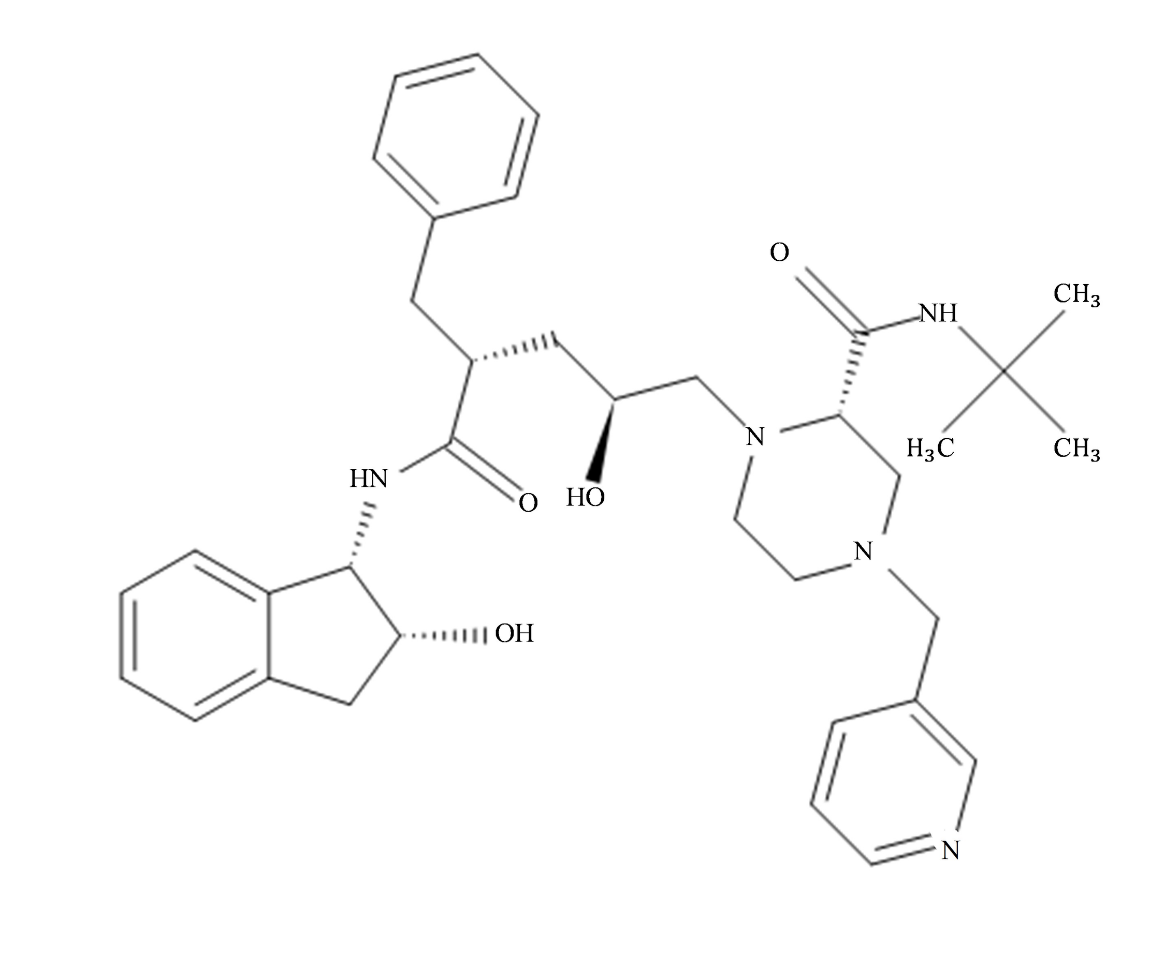
**

**Figure S7: Structure of Indinavir**

**

**

**Figure S8: Structure of Lopinavir**

**

**

**Figure S9: Structure of Paritaprevir**

**
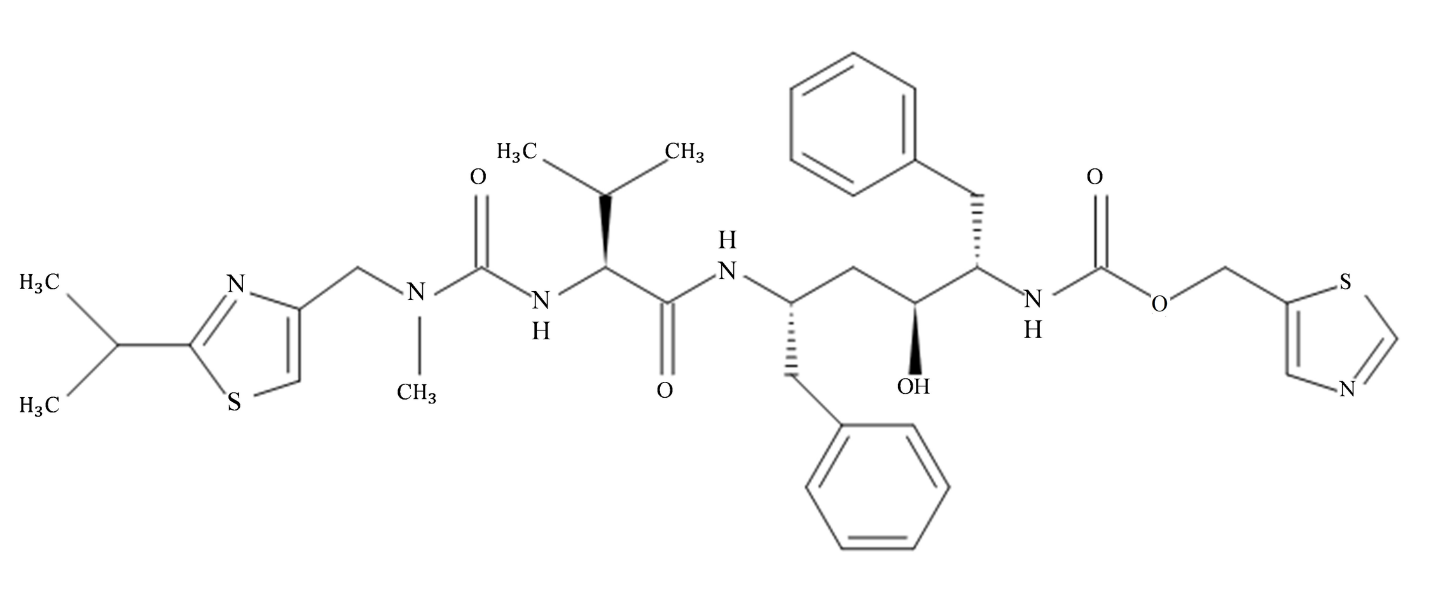
**

**Figure S10: Structure of Ritonavir**

**

**

**Figure S11: Structure of Saquinavir**

**

**

**Figure S12: Structure of Tipranavir**


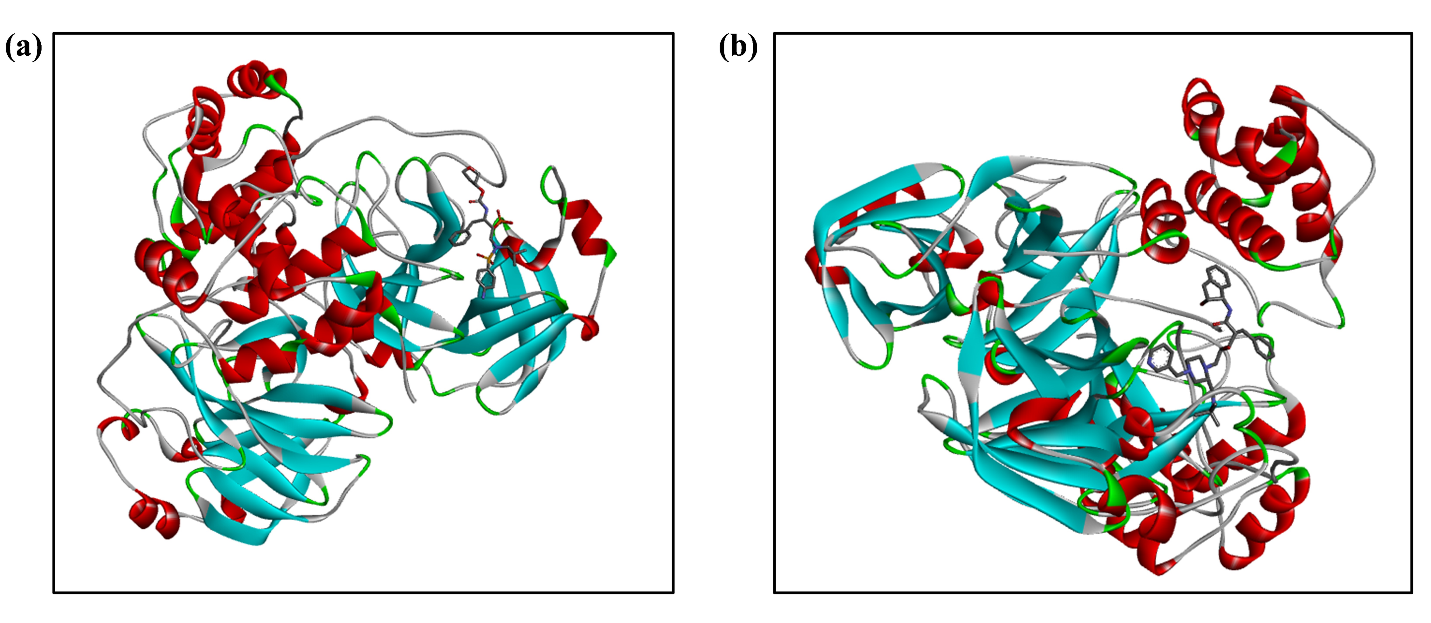


**Figure S13: Activity of protease inhibition of (a) Fosamprenavir and (b) Indinavir**


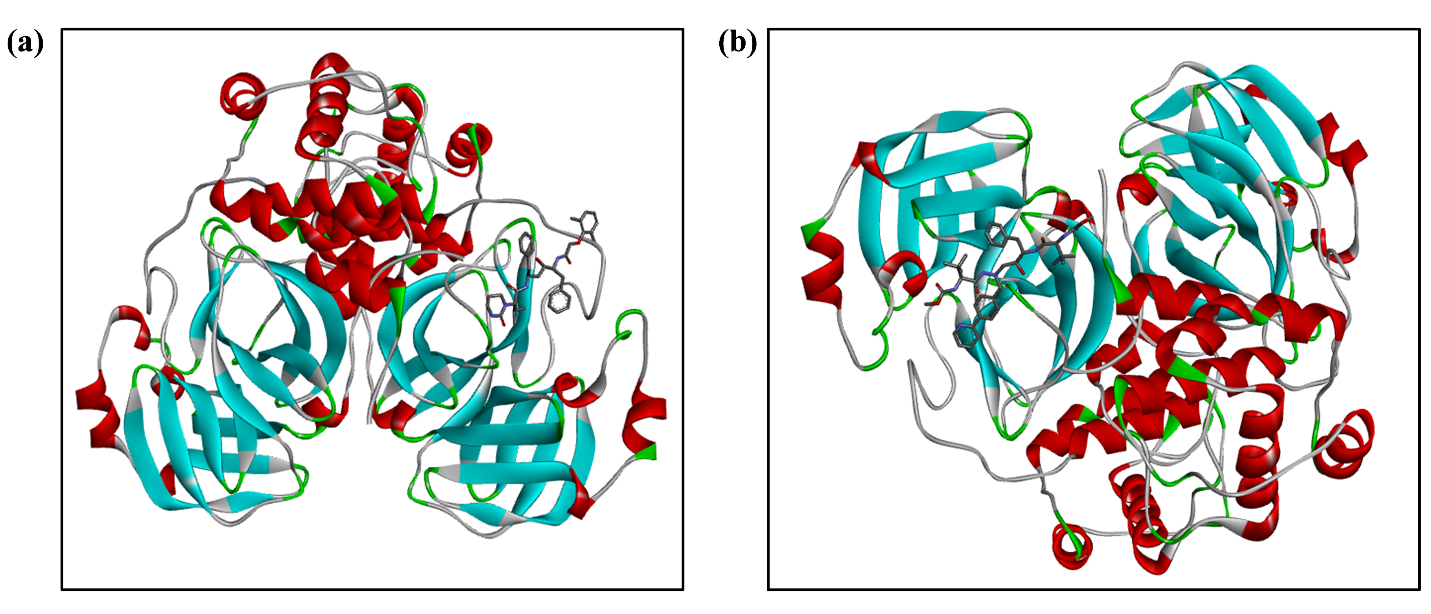


**Figure S14: Activity of protease inhibition of (a) Lopinavir and (b) Atazanavir**


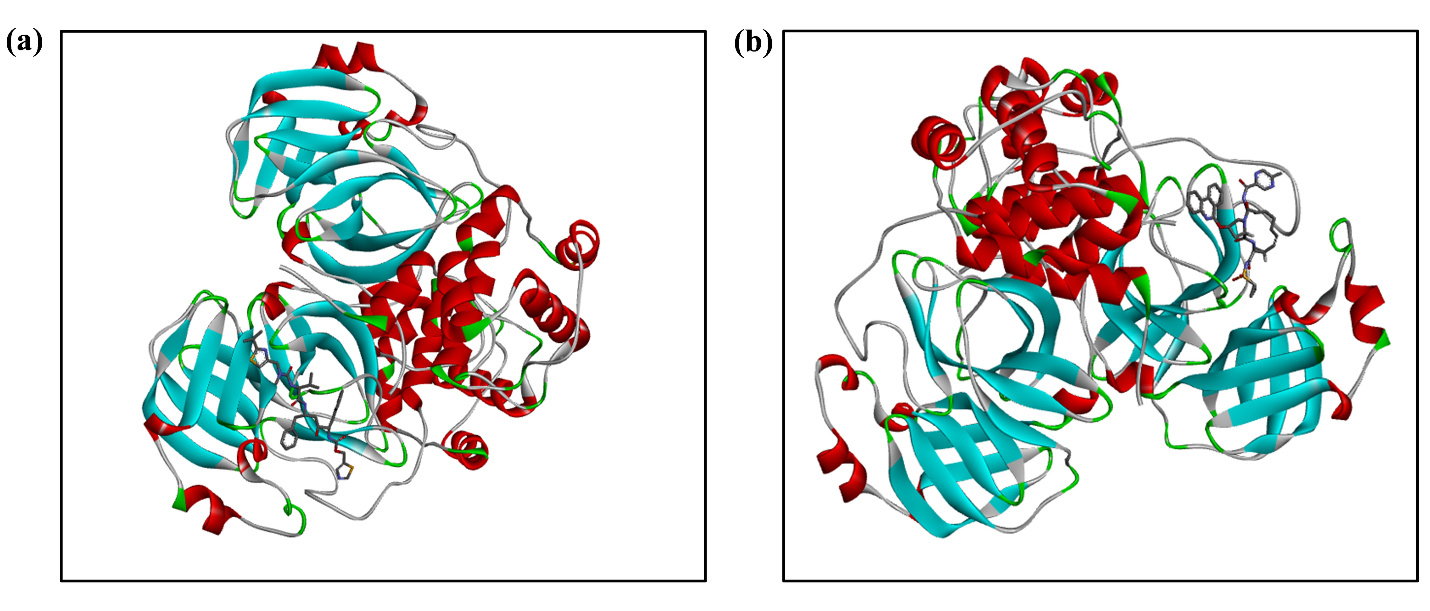


**Figure S15: Activity of protease inhibition of (a) Ritonavir and (b) Paritaprevir**


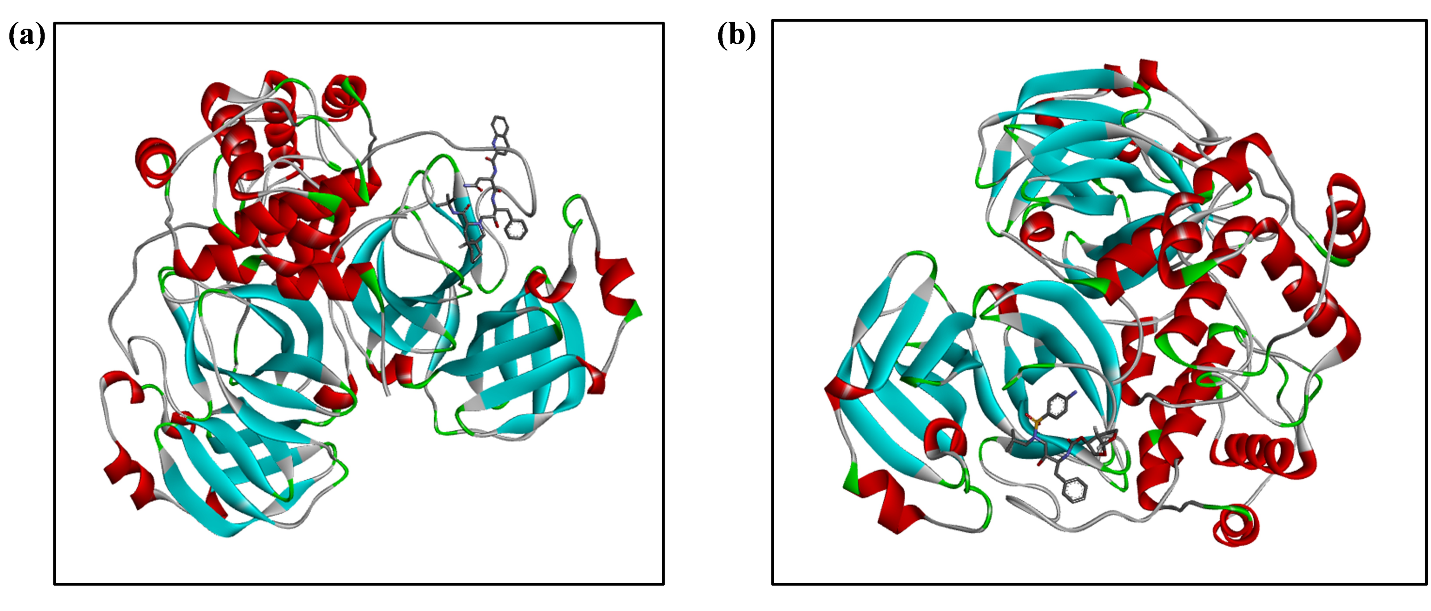


**Figure S16: Activity of protease inhibition of (a)** **Saquinavir and (b) Darunavir**


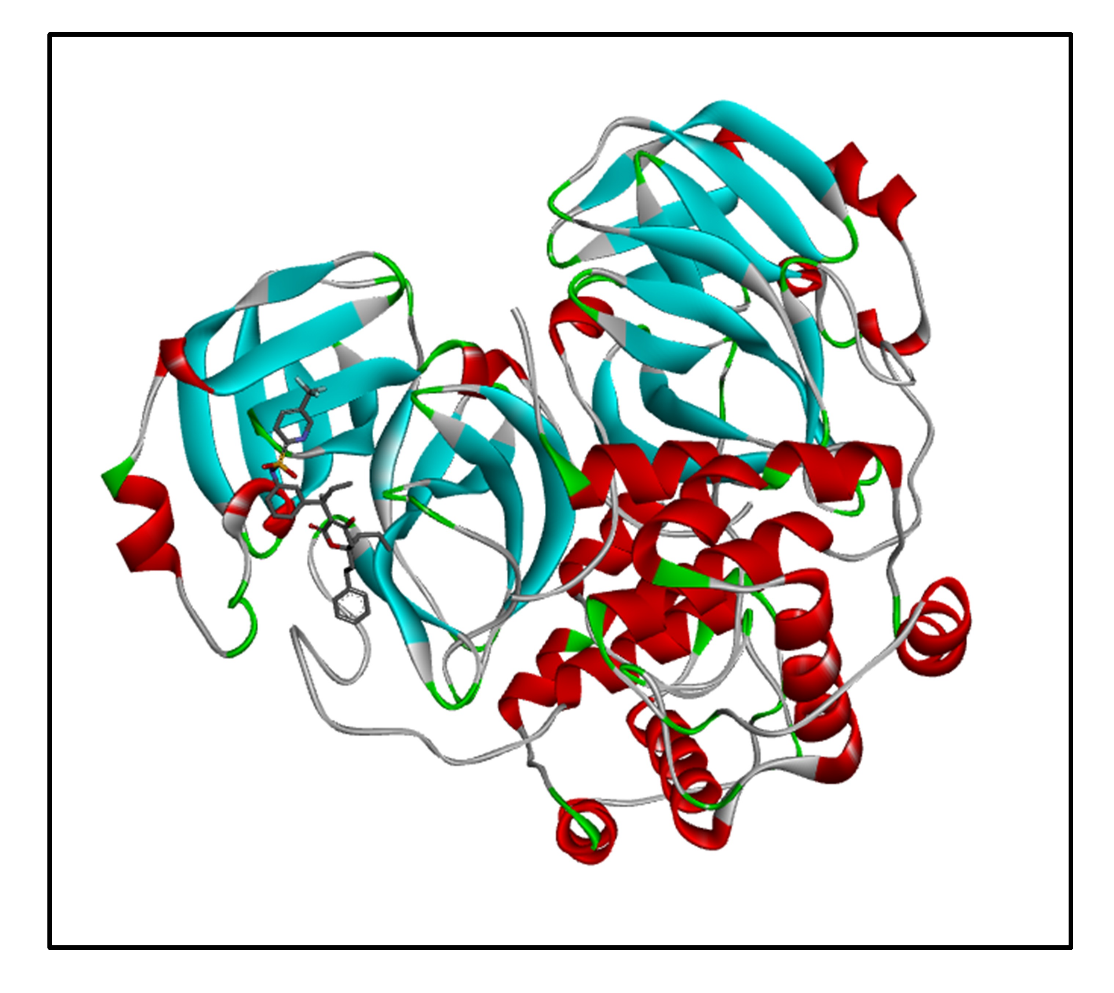


**Figure S17: Activity of protease inhibition of Tipranavir**
